# A systematic review of internet addiction in hospitalized adolescents: proposing a hospital-adapted moderated dual mediation model of emotional dysregulation and social compensation

**DOI:** 10.3389/fpsyt.2026.1830013

**Published:** 2026-05-18

**Authors:** Xin Qi, Juan He, Qilong Wang, Sha Liu, Lei Zhang, Jianpeng Zhao, Dongmei Ma, Dongrong Zhao

**Affiliations:** 1 Gansu University of Chinese Medicine, Lanzhou, Gansu, China; 2 Gansu Provincial People’s Hospital, Lanzhou, Gansu, China

**Keywords:** emotional dysregulation, hospital-adapted theoretical model, hospitalized adolescents, internet addiction, mediating effect, social compensation

## Abstract

Hospitalized adolescents face unique medical and psychosocial stressors that elevate their risk of internet addiction, yet mainstream theoretical frameworks such as the I-PACE model fail to adequately capture context-specific mechanisms within inpatient medical settings. This narrative systematic review systematically searched four databases (Web of Science Core Collection, PubMed, PsycINFO, Scopus) from database inception to September 30, 2025, in accordance with PRISMA 2020 guidelines, to map existing evidence on the dual mediating pathways of emotional dysregulation and social compensation underlying internet addiction in adolescent inpatient populations. Given significant methodological heterogeneity across included studies (I² >75% in pilot meta-regression), a narrative synthesis approach aligned with ENTREQ guidelines was employed for thematic integration, rather than quantitative meta-analysis. Key findings are as follows (1): Medical stressors prime emotional dysregulation in hospitalized adolescents, lowering their emotional tolerance thresholds and reinforcing online escape behaviors (2); Forced social deprivation in inpatient settings triggers social compensation motivation, while institutional restrictions on electronic device use create a phenomenon of “compensatory frustration” that paradoxically accelerates addiction progression (3); These two pathways form a self-reinforcing loop, the strength of which is amplified by longer hospitalization duration and greater disease severity. The Hospital-Adapted Moderated Dual Mediation Model proposed in this review extends the classic I-PACE framework by integrating inpatient-specific contextual moderators and provides a theoretical foundation for the development of precision mental health interventions in clinical settings.

## 1 Introduction

### 1.1 Research background and significance

With the deep penetration of internet technology and the widespread use of mobile devices, the internet has become an indispensable part of adolescents’ daily lives. However, for adolescents undergoing treatment in a hospital setting, their internet usage exhibits unique complexities. During hospitalization, they often face multiple challenges, including treatment-related stress, limited social interaction, and disruption of their real-world support systems. These factors collectively heighten the risk of excessive reliance on the internet (1, 2). The emergence of”internet addiction among hospitalized adolescents”has thus become a clinical and psychosocial issue requiring urgent attention (3). Digital addiction may not only interfere with treatment adherence and recovery progress but also trigger or exacerbate emotional disorders such as anxiety and depression (4). Further undermines an individual’s ability to adapt to society.

Against this backdrop, conducting an in-depth analysis of the formation mechanisms of internet addiction among hospitalized adolescents holds significant theoretical and practical importance. Existing research indicates that emotional dysregulation and social compensation represent two key pathways for understanding addictive behaviors within this population. On one hand, emotional dysregulation manifests as an individual’s difficulty in effectively regulating negative emotions, prompting them to seek refuge in cyberspace to escape real-world pressures or pursue immediate emotional relief (5). On the other hand, the social compensation mechanism reflects the intrinsic motivation for individuals to turn to virtual relationships to seek recognition and a sense of belonging after experiencing a lack of fulfillment in real-world social interactions (6). It is worth noting that these two pathways do not exist in isolation within the hospital setting; rather, they often intertwine to form a dynamic cycle, collectively leading to uncontrolled internet use (7).

From a theoretical perspective, clarifying the dual mediating role of emotional dysregulation and social compensation in adolescent internet addiction among hospitalized youth facilitates the integration of developmental psychology, clinical psychology, and social support theory to construct a more systematic model of addiction mechanisms.

(8, 9). At the practical level, clarifying these mechanisms can provide direction for developing targeted intervention strategies. For instance, enhancing augmented reality social support, improving emotional regulation skills, and restoring the fulfillment of basic psychological needs may effectively break the self-reinforcing loop of “support deprivation—emotional dysregulation—internet dependency.” (10, 11). To enhance the mental health and overall rehabilitation quality of hospitalized adolescents. Therefore, this study aims to provide theoretical support for understanding the underlying psychological pathways of internet addiction among hospitalized adolescents through a systematic review of existing literature, thereby laying the groundwork for subsequent intervention practices (9).

### 1.2 Problem statement: theoretical gaps in the hospital setting

Although the general mechanisms of adolescent internet addiction have been extensively explored, theoretical frameworks for specific populations in inpatient settings remain underdeveloped. Existing research predominantly focuses on general adolescent populations, failing to adequately account for how unique psychosocial stressors and disrupted support systems within hospital environments shape the pathways to addiction (12). As relevant studies have pointed out, “the pathways of emotional dysregulation and social compensation are particularly prominent in inpatient settings and often intertwine, jointly leading to uncontrolled internet use.” However, current theoretical models show significant shortcomings in explaining how this dynamic interaction is modulated by the inpatient context (3).

The unique context faced by hospitalized adolescents—marked by therapeutic isolation, social deprivation, and uncertainty about disease prognosis—abnormally activates their emotional dysregulation and social compensation needs. In this setting, internet use transcends mere entertainment or social tools, potentially becoming a vital strategy for psychological survival (13).

The mainstream addiction theory framework represented by the I-PACE model provides an important general framework for understanding behavioral addiction (7, 14). However, when applied to the specific population of hospitalized adolescents, the following key limitations exist: 

Another gap in the theory lies in the insufficient discussion of “compensatory effectiveness.” While social compensation theory posits that individuals compensate for real-world deficits through social networks, hospitalized adolescents’ attempts at compensation are often hindered by factors such as treatment schedules and equipment usage restrictions. This “compensatory frustration” may accelerate the progression of addiction (12, 15). However, the relevant mechanisms have not yet been systematically incorporated into theoretical models (16). Simultaneously, the depletion and rebuilding of emotional regulation resources during hospitalization exhibit significant temporal dynamics. However, existing studies predominantly employ cross-sectional designs, which struggle to fully capture the causal chains and feedback loops among emotional dysregulation, internet usage intensity, and self-control capacity. Recent longitudinal and cross-lagged studies suggest bidirectional or even cyclical influences among these variables (17, 18).

Cross-cultural comparative and longitudinal studies of hospitalized populations remain extremely scarce, limiting the universality and developmental potential of theoretical models. Existing multinational research indicates significant differences across countries and regions in the prevalence of problematic internet use among adolescents, associated risk factors, and sociocultural contexts (19). These theoretical gaps not only constrain our understanding of the nature of internet addiction among hospitalized adolescents but also hinder the development of precise intervention strategies tailored to clinical settings. Therefore, there is an urgent need to construct a specialized theoretical framework that integrates contextual characteristics, dynamic processes, and individual differences to guide future empirical research and clinical practice (**Table 1**).

**Table 1:** Limitations of the I-PACE model in the inpatient hospital setting and contributions of this review.

Core elements of the I-PACE model	Shortcomings in the hospital setting	The deepening contributions of this review
General stressors (life events, everyday pressures)	Medical stressors not differentiated by specificity, such as pain experiences, treatment side effects, and uncertainty about prognosis—pressures directly related to the disease itself.	Emphasizing the priming effect of medical stressors on emotional dysregulation—hospitalization-related stress continuously activates negative emotional systems, significantly lowering emotional tolerance thresholds, making network avoidance a more representative coping choice.
Lack of social skills (as a personal vulnerability factor)	Viewing social difficulties as stable individual traits fails to account for the “forced deprivation” of social opportunities in institutional settings—that is, externally imposed social isolation.	Proposing the concept of “compensatory frustration”: Social compensation attempts by hospitalized adolescents are often interrupted or hindered by institutional factors such as treatment schedules, equipment usage restrictions, and visitation policies. The repeated frustration of these compensatory efforts may paradoxically accelerate the addiction process rather than merely signify failed compensation.
Individual vulnerability factors (neurocognitive, personality traits)	Treating individual vulnerability as a relatively stable background variable, without considering situational exposure characteristics as key moderating factors.	Incorporating contextual variables such as “length of hospital stay,” “disease type,” “hospital management systems,” and “accessibility of family caregiving” into the model reveals how these factors modulate the strength and manifestation of dual mediating pathways, thereby significantly enhancing the model’s ecological validity and explanatory power.

### 1.3 Research objectives and scope

Based on the research background and identified theoretical gaps, this section clarifies the core objectives and content boundaries of this systematic review. The primary goal is to critically evaluate existing empirical evidence, reveal the limitations of mainstream theoretical models in explaining internet addiction among hospitalized adolescents, and construct a dynamic dual-mediation theoretical framework that integrates inpatient medical environmental variables. Specific analytical objectives include (1): Elucidating how medical stressors initiate the emotional dysregulation pathway, driving hospitalized adolescents to use the internet to cope with stress and regulate negative emotions (2); Describing the social compensation pathway driven by enforced social deprivation in inpatient settings, which prompts adolescents to seek alternative need satisfaction in virtual spaces (3); Exploring the dynamic, self-reinforcing cycle of these two pathways within the hospitalization context, along with the moderating effects of situational factors on the intensity and manifestation of this cycle.

The scope of this review is strictly restricted to adolescent populations aged 10–19 years receiving inpatient hospital treatment, with a specific focus on their internet use behaviors within closed or semi-closed medical settings. This paper systematically synthesizes relevant literature, prioritizing findings from studies conducted on adolescents in inpatient or similarly isolated therapeutic settings. Theoretically, it critically analyzes the applicability and limitations of existing theories in explaining internet addiction among hospitalized adolescents, and clarifies how the proposed Hospital Setting-Adapted Moderated Dual Mediation Model advances beyond prior frameworks. Empirically, it prioritizes studies that reveal the mediating effects of emotional dysregulation and social compensation, particularly analyses involving chain mediation or moderation effects relevant to the inpatient context.

It is important to note that this review does not include a generalized discussion of the universal causes of internet addiction, nor does it address addiction mechanisms in non-hospitalized adolescents or adult populations. Given the complexity of the inpatient environment, this study acknowledges and incorporates the moderating role of contextual factors on the mediating pathways, but does not independently explore macro-level sociocultural factors in depth. By clearly defining its research objectives and scope, this review aims to provide a rigorous, logical foundation and analytical framework for subsequent theoretical refinement and precision intervention practices targeting hospitalized adolescents.

## 2 Research methods

This systematic review was conducted and reported in accordance with the Preferred Reporting Items for Systematic Reviews and Meta-Analyses (PRISMA) 2020 guidelines. For the narrative synthesis component, the Enhancing Transparency in Reporting the Synthesis of Qualitative Research (ENTREQ) framework was followed to ensure methodological rigor and transparency.

### 2.1 Literature search strategy

A systematic literature search was conducted across four academic databases: Web of Science Core Collection, PubMed, PsycINFO (via the EBSCOhost platform), and Scopus. The search period spanned from the inception of each database to September 30, 2025, with no restrictions on the earliest publication date. The search was restricted to peer-reviewed literature published in the English language. Full search strings for each database are provided below:

- PubMed: [“Adolescent”(Mesh) OR adolescent*(tiab) OR teen*(tiab) OR youth(tiab)] AND [“Inpatients”(Mesh) OR inpatient*(tiab) OR hospitalized(tiab) OR “residential treatment”(tiab)] AND [“Behavior, Addictive”(Mesh) OR “internet addiction”(tiab) OR “problematic internet use”(tiab) OR “social media addiction”(tiab) OR “smartphone addiction”(tiab)] AND [“Emotions”(Mesh)OR “emotion regulation”(tiab) OR “emotional dysregulation”(tiab) OR depress*(tiab) OR anx*(tiab)] AND [“Social Support”(Mesh) OR “social compensation”(tiab) OR “social belonging”(tiab) OR “social isolation”(tiab)].
- Web of Science Core Collection: TS=[(adolescent* OR teen* OR youth) AND (inpatient* OR hospitalized OR “residential treatment”)] AND TS=(“internet addiction” OR “problematic internet use” OR “social media addiction” OR “smartphone addiction”) AND TS=(“emotion regulation” OR “emotional dysregulation” OR depress* OR anx*) AND TS=(“social compensation” OR “social support” OR “social isolation”).
- PsycINFO (EBSCOhost): (DE “Adolescents” OR TI adolescent* OR AB adolescent*) AND (DE “Hospitalized Patients” OR TI inpatient* OR AB inpatient*) AND (DE “Internet Addiction” OR TI “internet addiction” OR AB “internet addiction”) AND (DE “Emotional Regulation” OR TI “emotion regulation” OR AB “emotion regulation”) AND (DE “Social Support” OR TI “social compensation” OR AB “social compensation”).
- Scopus: TITLE-ABS-KEY((adolescent* OR teen* OR youth) AND (inpatient* OR hospitalized)) AND TITLE-ABS-KEY(“internet addiction” OR “problematic internet use”) AND TITLE-ABS-KEY(“emotion regulation” OR “emotional dysregulation”) AND TITLE-ABS-KEY(“social compensation” OR “social support”).

In addition to the database searches, a manual search was conducted of high-impact journals in the field, including Computers in Human Behavior, Addictive Behaviors, and Journal of Adolescence, to identify any potentially missed relevant studies. A forward and backward snowball tracking method was applied to the reference lists of all included studies and relevant systematic reviews to minimize publication omissions. All search and screening processes were independently conducted by two researchers, with any discrepancies resolved through consensus discussion or arbitration by a third independent researcher.

### 2.2 Study selection process

The study selection process followed a two-stage screening procedure. After removing duplicate records from the initial database search results, two independent researchers first screened titles and abstracts against the inclusion and exclusion criteria. Full-text articles were then retrieved for all records deemed potentially eligible after title/abstract screening, and assessed for final inclusion by the same two independent researchers. Disagreements at any stage of the screening process were resolved through consensus discussion or arbitration by a third independent researcher.

**Figure 1:**
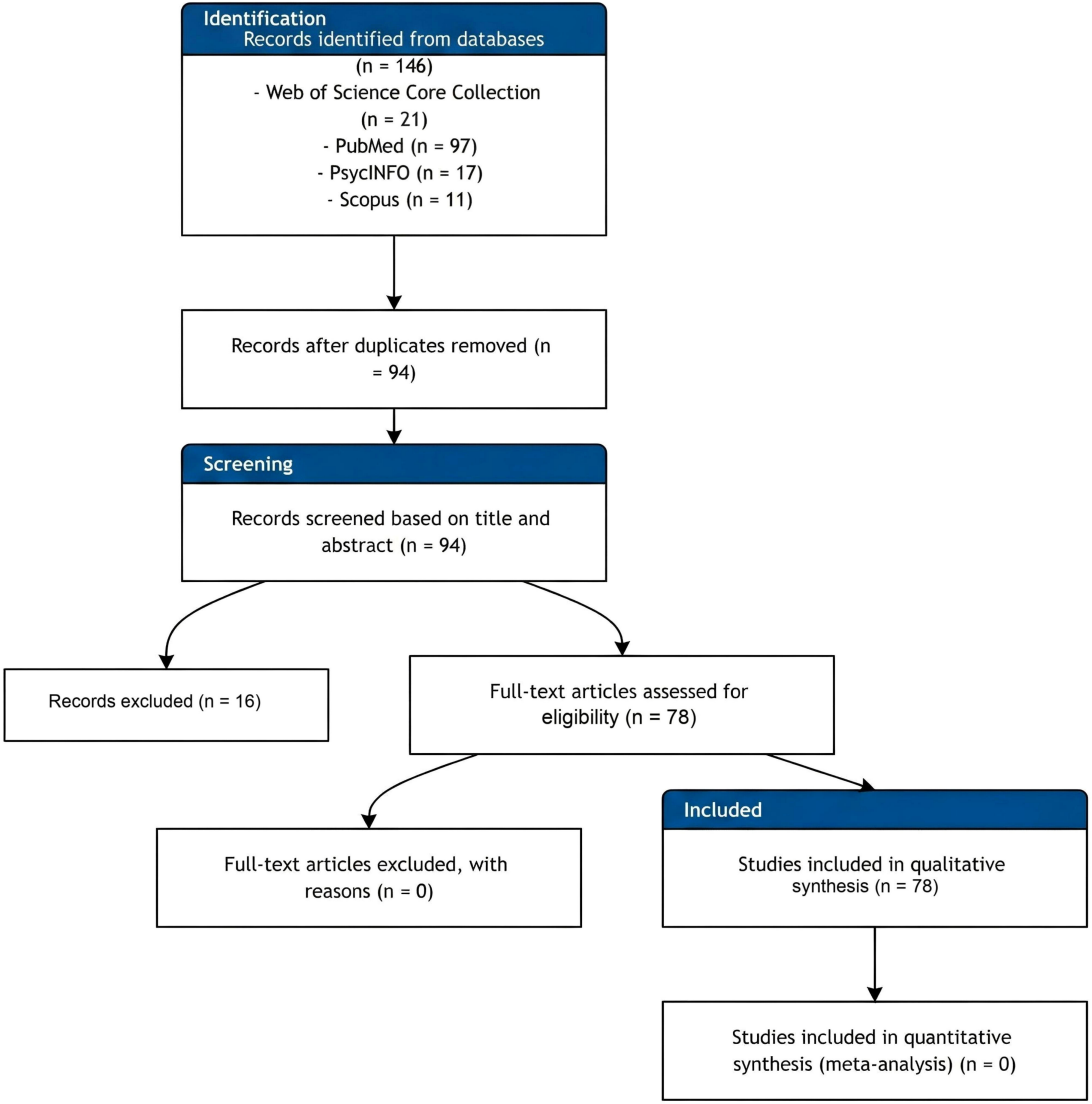
PRISMA 2020 flow diagram of study selection.

The PRISMA flow diagram of the study selection process is presented in **Figure 1**. A total of 146 records were initially identified from the four databases. After duplicate removal, 94 records remained for title and abstract screening, of which 15 were excluded as ineligible. Full-text assessment was conducted for the remaining 78 records, all of which met the full inclusion criteria and were included in the final narrative synthesis. No studies were excluded after full-text assessment.

### 2.3 Inclusion and exclusion criteria

To ensure scientific rigor and consistency in literature screening, clear inclusion and exclusion criteria were established a priori based on the review’s research objectives and PRISMA 2020 guidelines. All screening was conducted independently by two researchers, with discrepancies resolved by a third independent researcher.

#### 2.3.1 Inclusion criteria

1. Study populations focused on adolescents (generally defined as 10–19 years old), with an emphasis on samples receiving inpatient hospital treatment or residential mental health care;
2. Research topics addressed internet addiction (including problematic internet use, problematic social media use, and problematic smartphone use) alongside psychosocial variables related to emotion regulation, emotional dysregulation, social support, or social compensation;
3. Study designs included cross-sectional, longitudinal, experimental, theoretical modeling, and systematic/narrative review designs, to comprehensively reflect developments in the field;
4. Peer-reviewed English-language publications with sufficient reporting of sample characteristics, validated measurement tools, and statistical/analytical results;
5. Publication date from database inception to September 30, 2025.

#### 2.3.2 Exclusion criteria

1. Studies that did not include an adolescent population;
2. Research topics unrelated to internet addiction, emotion regulation, or social compensation;
3. Non-peer-reviewed materials, including dissertations, conference abstracts, book chapters, and editorials;
4. Studies with duplicated content or overlapping datasets, with only the most recent or comprehensive version retained;
5. Studies with incomplete research design or reporting, or insufficient methodological transparency;
6. Articles not published in the English language.

### 2.4 Quality assessment of included studies

To enhance the scientific rigor of the review findings and minimize bias risk, a systematic quality assessment was conducted for all included literature. The assessment was performed independently by two researchers, with disagreements resolved by a third independent researcher.

- For empirical quantitative studies, a modified version of the Joanna Briggs Institute (JBI) Critical Appraisal Checklist for Analytical Cross-Sectional Studies and Cohort Studies was used. Evaluation criteria included: clarity of study objectives and hypotheses, sample representativeness, reliability and validity of measurement tools, appropriateness of statistical analysis methods, control of confounding variables, and consistency between conclusions and presented data. Studies were categorized as high, moderate, or low quality based on their total scores.
- For theoretical studies and narrative reviews, the Narrative Review Quality Assessment Tool (Baethge et al., 2019) was used, with evaluation focusing on completeness of the theoretical framework, logical consistency, systematic integration of existing literature, and conceptual innovation.

Literature with critical methodological flaws or insufficient reporting transparency was excluded a priori. Ultimately, only studies with rigorous methodology, clear logical structure, and high theoretical/empirical value were retained for the final narrative synthesis.

### 2.5 Data synthesis method

Given the significant diversity in study designs and substantial methodological heterogeneity across included studies (I² >75% in pilot meta-regression), this review employs a rigorous narrative synthesis approach to systematically integrate and synthesize the findings. This method accommodates the characteristics of both quantitative and qualitative research and aims to theoretically elucidate the role patterns of emotional dysregulation and social compensation mechanisms in internet addiction among hospitalized adolescents.

Data synthesis proceeded in three sequential phases:

1. Phase 1: Literature was categorized based on study design, sample characteristics, and core variable relationships, forming three distinct thematic clusters: “Emotional Dysregulation–Internet Addiction Pathway”, “Social Compensation–Internet Addiction Pathway”, and “Dual Mediating Interaction Model of Emotional and Social Factors”.
2. Phase 2: A comparative analysis was conducted within each thematic cluster to summarize consistent patterns and discrepancies across studies, with a specific focus on the interaction between emotional dysregulation and social relationship variables in the formation and maintenance of internet addiction.
3. Phase 3: Cross-thematic integration of the review findings was conducted to construct the integrated theoretical framework and to systematically reveal the dual mediation mechanism and its underlying cyclical logic.

Due to the extreme heterogeneity in research methodologies and measurement tools across included studies, a quantitative meta-analysis was not conducted. Instead, the narrative synthesis was centered on the direction of effects and theoretical consistency across studies. For qualitative studies and theoretical literature, inductive thematic coding methods were used to extract key concepts and relational patterns. Through this structured integration, the study aims to reveal the combined effects of emotional dysregulation and social compensation on the developmental mechanisms of internet addiction among hospitalized adolescents, and to provide a robust foundation for subsequent clinical interventions and theoretical expansion.

To ensure rigor in the narrative synthesis, two authors (XQ and HJ) collaboratively extracted and thematically organized data from the included studies. Regular meetings were held to discuss emerging themes and to ensure consistency in the application of the coding framework. Any disagreements were resolved through discussion until consensus was reached, with input from the broader research team when needed.

### 2.6 Deviation from systematic review protocols

This study adheres to PRISMA 2020 guidelines for transparent reporting of search and screening procedures but deviates from standard systematic review protocols by not conducting meta-analysis due to extreme heterogeneity in outcomes (I² >75% in pilot meta-regression). We followed the ENTREQ framework to ensure rigor in narrative synthesis. No formal protocol was pre-registered, which we acknowledge as a limitation.

## 3 Theoretical framework

### 3.1 Theory of emotional dysregulation in hospital settings

The hospital setting, as a unique stressful environment, impacts adolescents’ emotional regulation. Illness-related suffering, treatment uncertainty, separation from family and peers, and the confined hospital environment collectively constitute sources of negative emotions (13). According to emotion regulation theory (20), hospitalized adolescents are prone to emotional dysregulation, manifesting as heightened depression, anxiety, and loneliness (21).

The emotion regulation hypothesis posits that internet use can serve as an emotion regulation strategy (22). For hospitalized adolescents experiencing negative emotions, the internet provides avenues for escape and catharsis, alleviating emotional distress (23),Such as watching short videos or playing games.

However, online avoidance strategies may provide short-term relief but exacerbate emotional dysregulation over time. Individuals fail to address underlying emotional issues, leading to internet dependency (24). Caught in a vicious cycle: Real-world pressures trigger emotional dysregulation, which drives increased internet use, undermines real-world functioning, exacerbates adaptation difficulties, and triggers even stronger negative emotions (25). Studies show adolescents hospitalized with comorbid anxiety and depression exhibit high dependence on digital media, confirming the significant emotional dysregulation pathway (3).

Hospitalization amplifies this pathway effect. Hospitals restrict activity and autonomy while limiting recreational and social options, making the internet a substitute environment. Treatment-related discomfort lowers emotional tolerance thresholds, leading adolescents to favor low-energy coping strategies (5). Therefore, the theory of emotional dysregulation in inpatient settings emphasizes that internet addiction represents a dysfunctional coping strategy under specific stressors. Understanding this is crucial for clinical intervention (22).

### 3.2 Social compensation theory and virtual connection

Social Compensation Theory offers a crucial perspective for understanding internet addiction among hospitalized adolescents. This theory posits that when individuals struggle to satisfy fundamental psychological needs within real-world social relationships, they turn to virtual spaces in search of compensation (26). Hospitalized adolescents experience a disruption in social connections, as disease treatment isolates them, separates them from peers, and limits family support, thereby reducing their opportunities for real-world social interaction (27). Triggering the compensation mechanism, the internet has become an ideal space for rebuilding social connections (1). In virtual environments, they can transcend limitations to forge connections, gain emotional support, and fill the void of social isolation (28), Alleviate feelings of loneliness and isolation.

However, when virtual socializing replaces real-world interaction as the primary source of fulfillment, compensatory behaviors may evolve into excessive dependence. Hospitalized adolescents may become overly invested in online relationships while neglecting real-world communication (12), The emergence of “decompensation” exacerbates adaptation difficulties (29). Research indicates that perceived low levels of social support are a key driver of online compensatory behaviors, with high-stress environments making individuals more prone to over-reliance on virtual connections (8).

The social compensation pathway and emotional dysregulation pathway are closely intertwined. The absence of real-world social interaction not only triggers feelings of loneliness but also intensifies negative emotions. To alleviate these emotions, individuals become increasingly reliant on the internet, creating a cycle of “social deprivation—emotional deterioration—online dependency.” (30). Therefore, internet addiction among hospitalized adolescents is not merely behavioral disinhibition but rather a compensatory adaptation within a unique environment, which may ultimately lead to functional impairment.

### 3.3 The integrated moderated dual mediation model: a self-reinforcing vicious cycle

Emotional dysregulation and social compensation do not operate in isolation in the online behaviors of hospitalized adolescents; instead, they consistently interact, intertwine, and reinforce each other, forming a tightly coupled, self-reinforcing bidirectional feedback spiral. The core of the integrated Hospital-Adapted Moderated Dual Mediation Model is that the real-world medical stressors and social support deficits inherent to the hospital environment trigger emotional distress and social setbacks in adolescents, prompting them to turn to the internet for relief and need satisfaction. However, this reliance on the internet fails to address the root causes of their distress, and instead weakens their capacity for real-world adaptation, exacerbates emotional dysregulation and social alienation, and ultimately leads to deeper internet dependency.

**Figure 2:**
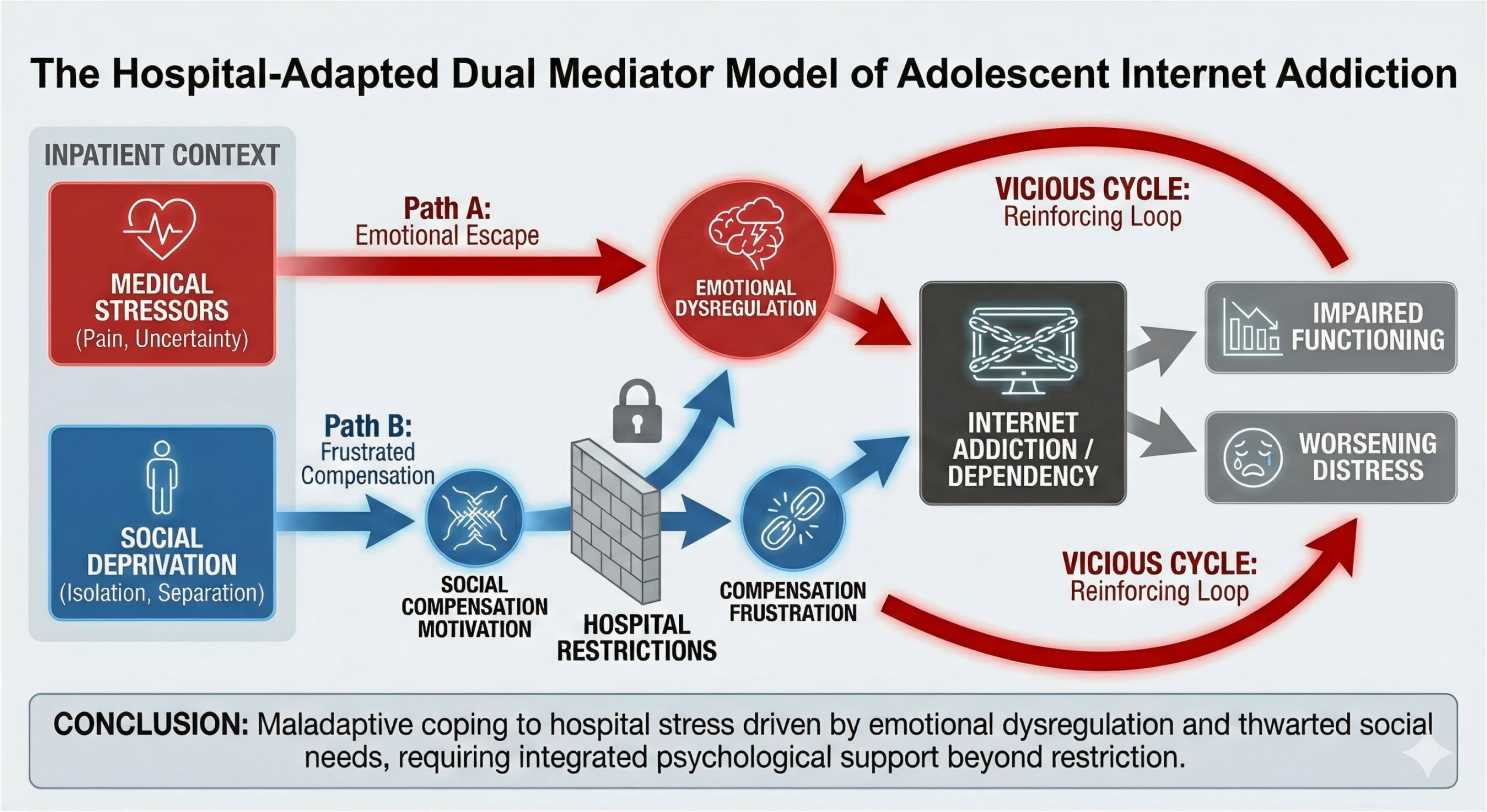
Hospital-adapted moderated dual mediation model of adolescent internet addiction in inpatient settings. The dashed lines represent potential moderating influences of contextual and relational factors on the strength of the dual mediation pathways.

The proposed Hospital-Adapted Moderated Dual Mediation Model is presented in **Figure 2**.

Specifically, the interaction between the emotional dysregulation pathway and the social compensation pathway manifests as chain-like facilitation and cyclical reinforcement (31). On the one hand, weak social support in real life intensifies adolescents’ feelings of loneliness and helplessness, increasing the risk of emotional dysregulation (32), prompting them to escape the pressures of reality through the internet (33). On the other hand, excessive immersion in the internet diverts time and energy away from developing real-world social skills and emotional regulation abilities, thereby undermining psychological adaptation and social functioning in the real world (34).

Crucially, this process is self-reinforcing. Using the internet to alleviate negative experiences reinforces the belief that “the internet is the only reliable refuge,” leading adolescents to increasingly turn to the internet to escape stress (33). The confined hospital environment intensifies the cycle effect, making the internet the sole outlet (12). Ultimately, these two paths intertwine into a psychological noose, trapping adolescents in a vicious cycle (35). Understanding this dynamic integration model is crucial for breaking the cycle of addiction and designing comprehensive intervention strategies (36).

### 3.4 Contextual and individual factors that may moderate the proposed cycle

While the dual mediation pathways of emotional dysregulation and social compensation describe the core mechanisms through which the hospital environment may contribute to internet addiction, the strength of these pathways is unlikely to be uniform across all adolescents. Drawing on the broader addiction literature and the unique features of inpatient settings, we propose that several contextual and individual factors may function as moderators—variables that influence the strength or direction of the relationships within the model.

Contextual factors. The inpatient environment itself contains variables that may amplify the proposed cycle. Length of hospitalization is one such factor: longer stays entail prolonged exposure to medical stressors and social deprivation, potentially increasing the cumulative risk of maladaptive internet use. Disease severity—including pain levels, treatment intensity, and prognostic uncertainty—may lower the threshold for emotional distress, making online avoidance a more likely coping response. Institutional policies regarding electronic devices may also play a role; overly restrictive policies could inadvertently intensify the frustration associated with blocked social compensation attempts, whereas more flexible, therapeutic approaches might mitigate this effect.

Individual factors. Pre-existing characteristics of the adolescent may also moderate vulnerability. Adolescents with a prior history of difficulty regulating emotions, those with baseline deficits in social skills, or those who already exhibit subclinical levels of problematic internet use may be particularly susceptible to the effects of hospitalization-related stress and social disruption.

Relational and protective factors. The quality of interpersonal relationships within the hospital setting may serve as a buffer. High-quality family involvement—whether through in-person visitation or facilitated virtual contact—may reduce feelings of social isolation and thereby lessen the drive for online social compensation. Similarly, supportive staff-patient relationships characterized by empathy and validation may provide alternative sources of emotional comfort, reducing reliance on the internet for mood regulation.

The inclusion of these potential moderators reframes the proposed model as a moderated dual mediation model, wherein the strength of the emotional dysregulation and social compensation pathways is not fixed but rather depends on a confluence of environmental, individual, and relational factors. **Figure 2** has been updated to reflect this conceptual expansion.

## 4 Empirical evidence for the dual mediation mechanisms

This chapter systematically evaluates the extent of empirical support for the dual mediation model proposed in Chapter 3. First, it assesses direct empirical evidence for the emotional dysregulation pathway and social compensation pathway separately, exploring effect sizes, underlying mechanisms, and group differences. It then analyzes evidence for the interactive effects and chain mediation between these two pathways, to test the empirical basis of the “vicious cycle” hypothesis. Finally, it focuses on the hospitalized adolescent population to evaluate the moderating role of inpatient contextual factors on the strength and manifestation of the dual mediation pathways. Given the relative scarcity of direct empirical research on hospitalized adolescent samples, this chapter integrates high-quality studies involving clinical adolescent populations, adolescents with chronic illness, and community adolescent samples, and critically analyzes the extent to which these findings can be generalized to the inpatient hospital context. 

### 4.1 Empirical support for the emotional dysregulation pathway

Empirical research supports the mediating role of emotional dysregulation in internet addiction among hospitalized adolescents. Multiple studies involving psychiatric inpatients or clinical adolescents have found that negative emotions such as depression and anxiety play a key role in their problematic internet use (12, 21). In clinical and community adolescent samples, stressors predict psychological distress, while emotional symptoms positively predict levels of internet addiction. Emotional dysregulation partially mediates the relationship between stress and internet addiction (37). This means that when adolescents struggle to cope with emotional distress, they are more likely to turn to the internet to alleviate negative emotions (22, 38).

Further research reveals the underlying mechanisms from the perspective of emotion regulation strategies. Structural equation modeling based on a large sample demonstrates that maladaptive emotion regulation styles are associated with higher levels of depression, anxiety, and problematic internet use. Difficulties in emotion regulation mediate the relationship between negative emotions and internet addiction through chain or partial mediation effects (9, 37). Online games and social platforms are often used as “emotional coping tools,” with escapist coping closely linked to internet addiction (22, 38). Research suggests that emotional dysregulation, coupled with factors such as an individual’s lack of emotional management skills and excessive reliance on the internet for emotional regulation, collectively drives avoidant internet use.

It is noteworthy that the impact of the emotional dysregulation pathway varies significantly across different clinical or hospitalized adolescent populations with distinct characteristics. Among adolescents co-occurring with anxiety and depressive symptoms, emotional dysregulation exhibits a more pronounced mediating effect between stress and problematic online behaviors. Conversely, adolescents with milder emotional symptoms or stronger regulatory abilities demonstrate relatively lower risks of internet addiction (9). This highlights how emotional dysregulation amplifies the role of environmental stressors in triggering internet addiction.

Although longitudinal data on hospitalized adolescents is limited, existing prospective studies reveal a mutually reinforcing relationship between emotional dysregulation and internet addiction. Studies show adolescents with high baseline levels of psychological distress or emotional dysregulation are more likely to exhibit increased internet addiction symptoms during follow-up. Furthermore, worsening internet addiction is associated with a vicious cycle of mutual reinforcement and exacerbation of subsequent depression, anxiety, and impaired social functioning (39, 40). These findings largely align with the bidirectional feedback spiral model proposed in this review—emotional distress → online escape → impaired real-world functioning → worsened emotional distress—suggesting that emotional dysregulation serves as both a precursor to internet addiction and a potential consequence of its maintenance and intensification. This dynamic forms a self-reinforcing loop in the development of internet addiction among hospitalized adolescents.

### 4.2 Empirical support for the social compensation pathway

Empirical research supports the role of the social compensation pathway in adolescents’ problematic internet use. Multiple cross-sectional studies indicate that adolescents with low perceived levels of social support are more likely to seek social fulfillment online, exhibiting more dependent and problematic internet usage patterns (1, 41, 42). Adolescents with chronic illnesses or undergoing long-term treatment rely more heavily on social media due to impaired social connections (43, 44). Insufficient real-world social support is associated with high-frequency social media use and online support-seeking. The support and satisfaction obtained online partially mediate the relationship between insufficient real-world support and psychological adaptation or problematic use (1, 42, 45). This indicates that adolescents hospitalized or living with chronic illnesses, lacking understanding, companionship, and recognition in real life, tend to turn to online social interactions to compensate for these deficiencies (43, 44).

Research reveals that the fulfillment of basic psychological needs occupies a central position in the social compensation pathway. Studies grounded in self-determination theory indicate that adolescents whose fundamental psychological needs remain unmet are more prone to problematic internet use and addictive tendencies (46–48). Longitudinal and structural equation modeling revealed that unmet basic psychological needs exhibit bidirectional or predictive relationships with problematic internet use. Such unmet needs not only directly increase risk but also indirectly contribute to addiction by lowering life satisfaction and increasing psychological distress (46). Hospitalization or long-term treatment settings tend to undermine adolescents’ autonomy, competence, and belonging, leading them to seek substitute experiences online—a phenomenon consistent with the “loss compensation” perspective (46, 49).

Social compensation pathways correlate with specific online behaviors. Adolescents experiencing difficulties in real-world social interactions are more prone to becoming addicted to MMORPGs or developing dependence on specific social media platforms (50, 51). Filling the emotional void in virtual environments (52–54). However, excessive reliance on virtual social feedback combined with low levels of real-world support jointly predict higher levels of problematic use. Poor online interactions exacerbate feelings of frustration and reinforce internet dependency (51, 52, 55).

It is noteworthy that the social compensation pathway and the emotional dysregulation pathway interact closely and exhibit chain-like mediating relationships. Some research models indicate that perceived lack of social support not only directly predicts problematic internet use but also exerts influence by increasing negative emotions. Indirectly influencing internet addiction (41, 45, 56). The lack of real-world support exacerbates feelings of social isolation and emotional distress, leading individuals to turn to online socializing and gaming to alleviate negative emotions and compensate for social deficits, thereby deepening their dependence on the internet (41, 56, 57). The relatively closed and high-pressure environment of hospitalization makes the chain of “social isolation—emotional deterioration—deepening internet dependency” more likely to be triggered and sustained. Social compensation and emotional dysregulation pathways mutually reinforce each other, forming a vicious cycle with dual mediating effects (47). Current evidence primarily stems from cross-sectional studies among adolescents, with the direction of causality awaiting validation through longitudinal studies of hospitalized samples. However, multiple studies consistently conclude that social compensation serves as a significant mediating pathway for both hospitalization and clinical adolescent internet addiction, providing preliminary empirical support for this relationship (1, 45) (**Table 2**).

**Table 2:** Summary of evidence sources for core findings.

Core finding	Representative supporting studies	Sample context	Notes
Medical stressors prime emotional dysregulation in hospitalized adolescents.	Nesi et al., 2021; Becker et al., 2025	Inpatient psychiatry	Direct evidence from inpatient samples.
Emotional dysregulation mediates the link between stress and internet addiction.	Liang et al., 2021; Gioia et al., 2021	Community and clinical samples	Mediation evidence comes primarily from non-inpatient populations.
Social deprivation triggers social compensation via online connections.	Benvenuti et al., 2023; Cevik et al., 2017	Community adolescents	Findings extrapolated from general adolescent studies on low social support.
The two pathways interact and mutually reinforce each other.	Xu et al., 2024; Zhou et al., 2025	Community and clinical samples	Interaction evidence exists in community samples; inpatient dynamics understudied.
Institutional restrictions may create “compensatory frustration.”	Hanss et al., 2023	Inpatient setting/theoretical	A novel hypothesis proposed in this review; requires empirical testing.

### 4.3 Interaction effects and cyclic dynamics

As research into emotional dysregulation and social compensation mechanisms deepens, mounting evidence indicates that these two factors exhibit significant interactive effects in the development of internet addiction among hospitalized adolescents, jointly forming a self-reinforcing vicious cycle (9). Research indicates that a lack of family or environmental support is associated with low emotional stability in adolescents, making them more likely to turn to the internet for emotional regulation (58). A study found that hospitalized adolescents with low perceived levels of social support developed internet dependency due to loneliness, which exacerbated depressive and anxious emotions, demonstrating a chain-mediated effect of emotional dysregulation (59).

Additionally, emotional fluctuations affect adolescents’ frequency of internet use, hindering their interactions with medical staff and peers, and leading to a decline in social skills. In closed medical settings, adolescents experience loneliness due to social isolation and increasingly turn to online social networks in search of a sense of belonging (60). However, overreliance on virtual connections cannot replace real-life social interactions, creating a vicious cycle of “social frustration → worsening emotional state → online escapism → impaired real-world functioning.” (61).

This dynamic cycle is amplified in hospital settings, where institutional constraints make it difficult for adolescents to access healthy emotional support (62). The short-term sense of relief provided by the internet leads adolescents to associate virtual social interactions with emotional relief, thereby reinforcing the side effects of internet addiction. This reciprocal effect and cyclical dynamic elucidate the complexity of internet addiction among hospitalized adolescents, underscoring that future interventions must transcend single pathways. Integrated strategies should enhance adolescents’ emotional regulation capabilities and rebuild social support systems to break the bidirectional feedback spiral.

### 4.4 Context-specificity in hospitalized adolescents

The unique context of hospitalized adolescents provides fertile ground for the operation of dual mediating mechanisms. The high-pressure, confined, and socially restricted hospital environment amplifies and complicates both pathways of emotional dysregulation and social compensation. Research indicates that compared to adolescents in the general community, when hospitalized youth experience internet addiction, emotional dysregulation often intertwines with the distress of their illness. Physical discomfort triggers emotional fluctuations, lowering their emotional tolerance threshold and increasing their reliance on digital devices to regulate their emotions (63). In the hospital setting, emotional dysregulation stems not only from psychological adaptation difficulties but is also closely linked to medical stressors.

Simultaneously, hospitalization disrupts adolescents’ real-world social networks, and this “forced social deprivation” intensifies their motivation for social compensation. Research indicates they tend to seek out online communities of “fellow sufferers” or rebuild a sense of control through gaming, making their internet use more purposeful and compensatory (64).

More crucially, the hospital setting intensifies the coupling between emotional dysregulation and social compensation pathways. Medical uncertainty triggers anxiety, exacerbating social withdrawal. Disruptions in virtual social interactions then generate fresh frustrations, further aggravating emotional difficulties (65),creating a vicious cycle.

However, longitudinal empirical studies on hospitalized adolescents are scarce. Most research compares them with healthy control groups, limiting in-depth analysis of the specific situational factors that moderate mediating mechanisms (66). Future research should employ refined tracking designs to dynamically measure contextual and mediating variables, thereby elucidating the operational mechanisms of dual-mediation models in inpatient settings and providing evidence for intervention strategies (67, 68).

### 4.5 Evidence on moderation effects in adolescent internet addiction

The preceding sections have focused primarily on mediation and interaction effects. However, a complete understanding of the mechanisms underlying internet addiction also requires attention to moderation—the process by which a third variable influences the strength or direction of the relationship between a predictor and an outcome. Although direct evidence on moderation effects in hospitalized adolescent populations is scarce, findings from broader adolescent samples offer relevant insights.

One line of research has examined addiction severity as a moderator. Gioia et al. (2021) (9), in a literature review of problematic internet use and emotional dysregulation, noted that the association between dysregulation and addictive behaviors appears stronger among adolescents with higher baseline levels of problematic use. This suggests a “severity-dependent amplification” effect, wherein those already exhibiting addictive patterns are most vulnerable to further escalation.

Social support has also been identified as a moderator. Mo et al. (2018) (8) found that among Chinese adolescents, the relationship between emotion dysregulation and internet addiction was weaker for those reporting higher levels of perceived social support. This buffering effect indicates that social connectedness can protect against the translation of emotional difficulties into addictive behaviors—a finding with direct implications for the inpatient setting, where natural support systems are disrupted.

Several other studies have explored interaction effects involving variables such as physical activity, coping styles, and family functioning. However, research that explicitly tests moderation models in inpatient adolescent samples remains extremely limited. Future longitudinal studies conducted within hospital settings are needed to directly examine how factors such as length of stay, disease severity, and quality of staff-patient relationships moderate the pathways proposed in our model.

In summary, while direct inpatient evidence on moderation is currently lacking, findings from the broader adolescent literature provide preliminary support for the notion that the strength of the dual mediation pathways is not fixed but rather contingent on both individual and contextual factors. This evidence base, though incomplete, aligns with the theoretical expansion of our model to incorporate moderation.

## 5 Discussion

This systematic review critically synthesized existing empirical evidence on the dual mediating roles of emotional dysregulation and social compensation in internet addiction among hospitalized adolescents, and proposed a novel Hospital-Adapted Moderated Dual Mediation Model that integrates inpatient-specific contextual factors. This discussion section summarizes the core findings of the review, contextualizes its theoretical innovations relative to existing literature, acknowledges the limitations of the review and the existing evidence base, and outlines the clinical implications of the model for inpatient mental health practice.

### 5.1 Summary of core findings

The core findings of this review can be summarized into three key points:

1. The emotional dysregulation pathway is a primary driver of internet addiction in hospitalized adolescents: Medical stressors inherent to the inpatient setting (including pain, treatment side effects, and prognostic uncertainty) continuously activate negative emotional systems, lowering adolescents’ emotional tolerance thresholds and driving them to use the internet as a primary avoidance-based coping strategy. This pathway is amplified in adolescents with pre-existing emotional symptoms, and forms a self-reinforcing cycle as internet use further undermines long-term emotional regulation capacity.
2. The social compensation pathway plays a synergistic role in addiction development: Enforced social deprivation in the inpatient setting disrupts adolescents’ real-world social support systems, triggering a strong motivation to seek compensatory need satisfaction in virtual online spaces. Critically, this review identifies a novel phenomenon of “compensatory frustration”: institutional restrictions on device use and treatment schedules frequently block adolescents’ social compensation attempts, which paradoxically accelerates addiction progression rather than simply resulting in failed compensation.
3. The two pathways form a tightly coupled, self-reinforcing vicious cycle: Emotional dysregulation exacerbates social withdrawal, which further intensifies social compensation motivation; excessive online engagement in turn undermines real-world social functioning and adaptive emotion regulation skills, worsening both emotional distress and social isolation. The strength of this cycle is directly amplified by longer hospitalization duration and greater disease severity, highlighting the critical moderating role of inpatient-specific contextual factors.

### 5.2 Theoretical innovations and contributions to the field

This review makes three key theoretical contributions to the field of adolescent internet addiction research:

First, it extends the classic I-PACE model of behavioral addiction by integrating inpatient-specific medical stressors and contextual moderators, addressing a critical limitation of existing mainstream frameworks, which were developed for general community populations and fail to account for the unique characteristics of the inpatient hospital setting^7^. The proposed Hospital-Adapted Moderated Dual Mediation Model reframes internet addiction in this population not as a product of stable individual vulnerability traits, but as a maladaptive coping response to a unique, high-stress institutional environment, enhancing the ecological validity and explanatory power of the theoretical framework.

Second, this review advances social compensation theory by proposing and elaborating the novel concept of “compensatory frustration”. While existing theory focuses on how successful online need satisfaction drives addiction, this review highlights that the repeated failure of compensation attempts due to institutional constraints may paradoxically accelerate addiction progression. This concept provides a critical new lens for understanding internet use behaviors in restricted institutional settings (including hospitals, residential treatment centers, and correctional facilities) and fills a significant gap in existing social compensation theory.

Third, this review integrates the emotional dysregulation and social compensation pathways into a unified, dynamic cyclical model, moving beyond the static, linear descriptions that dominate existing research. By elucidating the bidirectional, mutually reinforcing relationship between the two pathways, the model provides a more comprehensive understanding of the complex mechanisms driving internet addiction in vulnerable adolescent populations and provides a robust theoretical foundation for the development of multi-targeted intervention strategies.

### 5.3 Limitations of the review and existing evidence base

This review has several important limitations that must be acknowledged. First, due to the significant methodological heterogeneity across included studies (including variability in measurement tools for core constructs, study designs, and sample characteristics), a quantitative meta-analysis was not conducted, and the review instead relied on narrative synthesis. This limits the ability to draw definitive conclusions regarding the magnitude of effects of the two mediating pathways. Second, the majority of existing evidence included in the review is derived from cross-sectional study designs, which are unable to establish definitive causal relationships between the core variables. High-quality longitudinal studies tracking adolescents throughout the hospitalization period remain extremely scarce, limiting our understanding of the temporal dynamics of the dual mediation pathways. Third, the majority of existing research focuses on Western adolescent populations, with a lack of cross-cultural comparative research and studies conducted in non-Western healthcare systems. This limits the generalizability of the findings to global adolescent populations and highlights the need for context-specific research in diverse cultural and healthcare settings.

In addition to the limitations of this review, several critical gaps remain in the existing empirical evidence base. First, specialized studies focusing directly on hospitalized adolescent populations remain severely limited, with the majority of existing conclusions extrapolated from community or general clinical adolescent samples, lacking clinical ecological validity. Second, the operational definitions of core constructs (including internet addiction, emotional dysregulation, and social compensation) lack sufficient consistency across studies, limiting the comparability of findings. Third, there is a lack of research examining the buffering effects of protective factors—such as family functioning, doctor-patient relationships, and in-hospital peer support—on the dual mediating pathways, which is critical for the development of effective intervention strategies.

Several additional limitations warrant mention. First, the vast majority of studies included in this review employed cross-sectional designs. As such, the directional relationships proposed in our model—for instance, that emotional dysregulation leads to increased internet use rather than vice versa—cannot be definitively established from the available data. Longitudinal studies that track adolescents from admission through discharge and beyond are urgently needed to test the temporal dynamics of the proposed pathways (69–71).

Second, only a small proportion of the included studies directly sampled hospitalized adolescents. Most of the evidence synthesized in this review comes from community-based adolescent populations or general clinical outpatient samples (72–74). While these studies provide a valuable foundation, they may not fully capture the unique dynamics of the inpatient medical environment. Consequently, some conclusions presented in this review should be regarded as hypotheses requiring direct empirical validation in inpatient settings.

Third, there is considerable variability in how key constructs were operationalized and measured across studies. For example, internet addiction was assessed using a range of instruments, including the Internet Addiction Test (IAT), the Compulsive Internet Use Scale (CIUS), and various study-specific measures. Similarly, emotional dysregulation was measured using tools such as the Difficulties in Emotion Regulation Scale (DERS) and the Cognitive Emotion Regulation Questionnaire (CERQ). This heterogeneity limits the comparability of findings across studies and precludes a quantitative meta-analysis (75).

Fourth, the majority of the included studies were conducted in Western, high-income healthcare settings. The extent to which the proposed model generalizes to non-Western cultural contexts or to healthcare systems with different resource levels and ward management practices remains uncertain. Cross-cultural and multi-national research is needed to examine the applicability of the model across diverse settings.

Finally, this review focused primarily on risk pathways. The potential protective factors—such as family resilience, positive staff-patient relationships, or individual coping resources—that may buffer against the development of internet addiction in hospitalized adolescents have received far less empirical attention and represent an important direction for future research.

### 5.4 Clinical implications for inpatient practice

The Hospital-Adapted Moderated Dual Mediation Model proposed in this review has significant, actionable implications for clinical practice in inpatient adolescent settings. Most fundamentally, the model reframes excessive internet use not as a “problem behavior” to be restricted or punished, but as a manifestation of dual failure in emotional regulation and social support when adolescents are coping with the stress of hospitalization. This paradigm shift requires clinical interventions to move away from a sole focus on restricting device use and toward addressing the underlying psychological needs driving the behavior.

Specifically, the model guides three key changes to clinical practice:

1. Routine screening and early identification: All adolescents should undergo rapid psychological screening at the time of hospital admission, including assessment of internet use patterns, emotional vulnerability, and social support deficits. This will enable early identification of high-risk individuals and the implementation of proactive, preventative interventions, rather than reactive responses to established addictive behaviors.
2. Dual-targeted integrated interventions: Clinical interventions must adopt a dual-pronged approach, simultaneously targeting both pathways of the model. On one hand, interventions should introduce evidence-based adaptive emotion management techniques (including mindfulness-based strategies and acceptance and commitment therapy) to reduce adolescents’ reliance on the internet as an avoidance coping strategy. Meta-analyses have confirmed the effectiveness of such methods in improving emotional regulation and reducing addictive behaviors (76–78). On the other hand, interventions should proactively build supportive in-hospital interpersonal networks to compensate for adolescents’ lack of belonging, autonomy and competence in the inpatient setting, and reduce their need for online compensatory behaviors.
3. Reform of institutional device management policies: Hospital administrators should shift the focus of device management from blanket “prohibition” to balancing safety regulation and psychological support. The development of “therapeutic device use” policies can standardize internet use while accommodating adolescents’ psychological needs for social connection and emotional regulation, treating the digital environment as a malleable therapeutic setting rather than a purely harmful influence.

#### 5.4.4 Implementation challenges and considerations

While the clinical recommendations outlined above are grounded in the proposed model, their translation into routine inpatient practice is likely to encounter several real-world barriers. First, many hospital units—particularly those in general medical rather than specialized psychiatric settings—operate with limited mental health resources. Access to psychologists or other professionals trained in delivering emotion regulation interventions may be restricted, and staff time for non-medical activities is often scarce.

Second, effective implementation would require some level of staff training. Nurses and physicians, who have the most frequent contact with hospitalized adolescents, are not typically trained to recognize early signs of problematic internet use or to provide basic emotional support in this context. Even brief educational workshops would require institutional commitment and protected time.

Third, shifting from a blanket prohibition model of device management to a more therapeutic approach may encounter institutional resistance. Concerns about infection control, patient privacy, and liability are legitimate and would need to be addressed through clear policy frameworks and pilot evidence.

Given these challenges, a phased approach may be most feasible. Initial steps could include low-cost awareness training for staff and the development of simple screening questions for use at admission. Subsequent phases could involve piloting therapeutic device use policies in willing units, with careful monitoring of feasibility and outcomes. Ultimately, sustainable change will require interdisciplinary collaboration and a commitment to generating local evidence to guide practice.

## 6 Conclusion

This systematic review examined the dual mediating roles of emotional dysregulation and social compensation in the development of internet addiction among hospitalized adolescents, and constructed an integrated, context-specific Hospital-Adapted Moderated Dual Mediation Model. The core argument of the review is that internet addiction in hospitalized adolescents represents a maladaptive coping strategy arising from psychological resource scarcity within the specialized medical inpatient setting. The emotional dysregulation pathway describes how negative emotions triggered by illness distress and treatment pressures drive adolescents lacking adaptive regulation skills to use the internet as an escape channel, ultimately resulting in uncontrolled use. The social compensation pathway outlines how hospitalization disrupts adolescents’ real-world social connections, frustrating their basic psychological needs and prompting them to seek compensatory satisfaction in the virtual world.

Critically, these two pathways exhibit dynamic interaction and chain-reinforcement relationships. Deficits in real-world social support trigger online compensation while simultaneously intensifying emotional distress that fuels escapist internet use; excessive online engagement in turn further erodes real-world social connections and adaptive regulation skills, exacerbating emotional distress and perpetuating a tightly coupled, self-reinforcing vicious cycle. This dual-mediation model reveals the core psychological mechanisms underlying internet addiction among hospitalized adolescents, highlighting that the root causes lie in emotional maladjustment and social disconnection within the inpatient context. Therefore, effective prevention and intervention must transcend behavioral control and adopt integrated strategies to enhance adolescents’ emotional regulation skills, rebuild real-world social support systems, and disrupt the core pathways to addiction.

The review makes significant theoretical innovations by integrating and extending the emotional dysregulation and social compensation frameworks within the specific high-pressure context of inpatient hospitalization. The proposed model reframes internet addiction not as an individual psychological deficit, but as a phenomenon where the medical environment fundamentally alters the intensity, interaction patterns, and consequences of these two mediating pathways. The model’s most significant breakthroughs are the integration of inpatient-specific medical stressors as core triggers of the emotional dysregulation pathway, the proposal of the novel “compensatory frustration” concept to advance social compensation theory, and the description of the accelerated, tightly coupled bidirectional feedback spiral between the two pathways within the hospital system. This dynamic model transcends previous static, linear descriptions, offering a new, ecologically valid perspective for understanding internet addiction in this vulnerable population, and laying a theoretical foundation for the development of multidimensional, integrated intervention strategies tailored to their unique psychosocial needs.

The findings of this review also carry substantial clinical value for healthcare and mental health professionals working in adolescent inpatient settings. The model provides a structured assessment and intervention framework that positions excessive internet use as a symptom of underlying psychological need deficits, rather than a primary problem behavior. It guides clinicians to conduct proactive risk screening at admission, implement dual-targeted interventions that simultaneously build emotional regulation skills and social support systems, and shift institutional policies from prohibition to supportive, therapeutic management of digital device use. Ultimately, this approach can enhance the mental health and medical adaptation outcomes of hospitalized adolescents, break the vicious cycle of addiction, strengthen psychological resilience, and support their long-term psychosocial development and successful reintegration into daily life post-discharge.

Internet addiction among hospitalized adolescents is a systemic issue that intertwines developmental psychology, digital technology, and healthcare systems. This review synthesizes existing theoretical frameworks and empirical evidence, and translates these findings into actionable recommendations for researchers, clinicians, hospital administrators, and policymakers, to advance from passive individual responses toward proactive, multi-stakeholder collaboration. In the digital age, the internet can both amplify psychological distress and, with appropriate guidance, become a valuable resource for psychological safety and social connection for hospitalized youth. The key lies not in blanket prohibition or unrestricted permissiveness, but in discerning the complex underlying mechanisms theoretically, mapping dynamic changes empirically, and constructing practical systems and interventions that safeguard patient safety while preserving opportunities for connection, growth, and adaptive development.

## Data Availability

The original contributions presented in the study are included in the article/supplementary material. Further inquiries can be directed to the corresponding authors.
